# Assessment of information resources for people with hypodontia

**DOI:** 10.1038/bdjopen.2018.1

**Published:** 2018-03-09

**Authors:** Sophy Barber, Sue Pavitt, David Meads, Balvinder Khambay, Hilary Bekker

**Affiliations:** 1 Orthodontic Department, Leeds Dental Instsitute, Clarendon Way, Leeds, UK; 2 School of Dentistry, Level 6, Worsley Building, Clarendon, Way, Leeds LS2 9LU, UK; 3 Leeds Institute of Health Science, Level 10, Worsley Building, Clarendon, Way, Leeds LS2 9LJ, UK

## Abstract

**Aim:**

To assess the adequacy of patient information to support understanding and decision-making for people affected by hypodontia.

**Methods:**

1) Questionnaire to understand the provision of patient information by dentists; 2) Systematic search to identify online open-access patient information; 3) Quality assessment of written patient information.

**Results:**

Questionnaire response rate was 49% (319/649); 91% examined and/or treated people with hypodontia. Most general dentists referred patients to specialist services without providing written hypodontia information. The majority of dental specialists provide patient leaflets but less than a third used web-resources. Only 19% of respondents felt current resources were fit-for-purpose. Thirty-one patient resources (18 leaflets and 13 online) were assessed against quality criteria. The aim of the resource was seldom explicit, the content was often incomplete and variation in readability scores indicated high levels of literacy were required.

**Discussion:**

Access to, and quality of, patient information for hypodontia is inadequate. Current resources are not sufficiently comprehensive to prepare young patients to engage in shared dental care decisions with their parents and/or dental professionals.

**Conclusion:**

There is a need for improved access to, and provision of, information about hypodontia if dental professionals want to meet best practice guidance and involve patients in shared decision-making.

## Introduction

Hypodontia is a life-long condition that usually presents in early adolescence with variation in presentation, severity and impact. Determining an appropriate care plan with young patients and their parents can be challenging due to variability in the condition and treatment, and changes in understanding that arise when children transition into young adults.^1^ Dental professionals need to consider individual patient values, health perception, attitudes and behaviour to agree and implement effective treatment. Information should help patients and parents to understand the dental problems and treatment options, and enable dental professionals to elicit what is important to patients before gaining informed consent.^2,3^ However, patient information in dentistry has been shown to have a number of shortcomings.^4–10^ The majority of studies used reading grade assessment tools alone or in combination with a quality assessment measure, such as the DISCERN tool, a questionnaire for systematically assessing health information. The studies found inadequacies in the stated aim of the resource, the comprehensiveness and accuracy of content, transparency around sources, signposting to further information and support, and readability. This will potentially limit its capacity to improve patient’s ability to obtain, read, understand and use dental care information to make appropriate health care decisions, described as dental health literacy.

Information resources in dentistry aim to improve dental health literacy by (a) informing, preparing and educating patients, (b) enabling more effective engagement with dental professionals and service delivery, and (c) encouraging participation in treatment decision making and/or self-management.^11–13^ Evidence from the psychological sciences has been used to improve the efficacy of patient resources by providing understanding about how information is used to make treatment decisions and how people can be encouraged to consider their own values and make trade-offs in the context of their lives. Co-development and testing of resources with users has been shown to be highly effective in developing patient information in other health settings.^14^

Patient Information Leaflets (PILS) remain the most common source of patient information and are largely produced by care providers such as hospital trusts and dental practices, charities and professional bodies such as the British Orthodontic Society and by industry, such as manufacturers of dental implants. PILs have been shown to increase knowledge, treatment adherence, informed decision-making, and to reduce distress and anxiety.^15,16^ More recently online information has gained popularity and orthodontic patients report searching Google, Wikipedia, NHS Choices and the British Orthodontic Society website for information about treatment options and implications.^17^ Online resources allow patients to access information without seeking dental professional advice but it is unclear if online resources provide information that supports good decision-making about dental services and care. Previous studies assessing the quality of online patient resources for dental procedures identified high variability in quality and readability.^8–10^ Patients are increasingly using social media to seek information, share experience and look for support. This shift in accessing information provides opportunities for rapid dissemination of up to date information but also presents new challenges for quality assurance.

The aim of this research was to assess the adequacy of patient resources to support understanding of, and decision-making about, hypodontia treatment. The objectives are:

- To investigate general and specialist dentists’ use and views of patient information resources.
- To identify the availability of patient information resources (leaflets and online resources) about hypodontia and its treatment.
- To evaluate the quality of resources about hypodontia for people affected by hypodontia.

## Materials and methods

### Design and sample

Mixed methods were used:

1. A questionnaire for general dentists and three specialist dental groups about their use and views of patient resources for hypodontia. Except where indicated, all members of each group were invited from: the Ectodermal Dysplasia Society list, which lists clinic where hypodontia is managed (*n*=20); British Orthodontic Society list (*n*=145); General Dental Council list of Specialist Paediatric Dentistry (*n*=218); British Society of Restorative Dentists (*n*=86); British Society of Prosthodontists (*n*=6); General Dentist Practitioners in West Yorkshire Area Team list (174 selected from ~350). The variation in numbers is due to the number of members identified by each source.
2. A systematic search using three Internet search engines (Google, Bing, Yahoo) was carried out on 14 February 2016 to identify online resources for people affected by hypodontia. Search terms were hypodontia, developmentally or congenitally missing teeth, absent teeth, tooth/dental aplasia/agenesis. Inclusion criteria for websites included: information about hypodontia and its treatment; for patients and families; English; >500 words to enable quality assessment. Exclusion criteria for websites were: other causes for missing teeth; aimed at health professionals; marketing resources.
3. A content analysis of leaflets and online resources about hypodontia. All eligible written patient information identified by questionnaire and the Internet search were submitted for quality assessment.

Ethical approval was obtained from the University of Leeds Dental Research Ethics Committee on 12April 2016 prior to commencement of the study (050216/SB/188).

### Materials and procedure

Using publically available addresses, each dental professional was sent information about the study with the questionnaire and a stamped addressed envelope to return any written patient information resources. No reminders were sent because this was judged to significantly increase the time and costs of the research. Instead, use of a personalised letter and inclusion of a stamped return envelope were chosen as means to increase the response rate. The questionnaire was developed by the authors and piloted with seven dentists (one Consultant Orthodontist, one Post-CCST Registrar in Paediatric Dentistry, two Specialty Registrars in Restorative Dentistry and three general dentists) for ease of completion and understanding (contact authors for a copy). The questionnaire assessed:

- Demographics: name, job title, speciality, role in examining and treating hypodontia.
- Provision of hypodontia information: verbally, leaflet, internet resource.
- Views about written patient information:
- Would having information for patients about hypodontia be useful? (Yes/No)
- Do you think current sources of information for patients with hypodontia are fit for purpose? (Yes/No/Uncertain)
- What would be your preferred method for providing patients with information about hypodontia? (Face-to-face/Leaflet/Internet-written/Internet-video)
- Would you direct patients to a web-based information resource if the information were good quality? (Yes/No)
- How do you feel the information could be improved? (Free text)

Copies of leaflets and website details were requested; identified resources were recorded in Microsoft Excel 2016 and submitted for quality assessment.

The systematic Internet search was completed using recommended environmental scan methods.^18^ The search terms were entered successively into each search engine by SB. The first 10 pages of each search, which included 100 hits, were screened for websites (Supplementary Table 1). Those meeting the inclusion criteria were ‘bookmarked’ in the web browser at the time of searching and recorded in Microsoft Excel 2016. Websites were reviewed for suitability against the eligibility criteria by two reviewers (SB and HB) in duplicate. All online resources that were judged to be relevant were included and submitted for the quality assessment. Targeted website searching was completed for the following organisation: NHS Choices, Department of Health, Royal College of Surgeons, British Dental Association, British Dental Health Foundation, British Orthodontic Society, British Society of Paediatric Dentistry, British Society of Restorative Dentistry, Restorative Dentistry UK, Association of Dental Implants.

Quality assessment was undertaken for all resources identified through the questionnaire and for relevant online resources. Assessment was conducted independently and in duplicate by two reviewers (SB and HB) and agreement on the final score was reached by consensus. For pragmatic reasons, authors of the resources were not contacted to establish whether patient input was sought during development. The Flesch-Kincaid Grade Level and the Simple Measure of Gobbledegook (SMOG) were used to assess readability levels using an online tool (www.online-utility.org). The quality assessment criteria was developed using guidance from DISCERN,^19^ IPDAS^20^ and tools from similar studies.^8,12,21^ A data extraction form was used to record:

- Demographics: publisher, year.
- Design features aiding literacy: style, text layout, use of images, literacy aids, usability, and accessibility.
- Content: purpose, condition, treatment options, treatment consequences and risks, health service, value clarification, decision making components.
- Quality judgment: accuracy, transparency, conflict of interest.

### Analysis

Descriptive data analysis was undertaken to address the following research questions:

- How do different dentist groups use information resources for people affected by hypodontia?
- How useful do dentists perceive current patient resources to be?
- Do existing patient resources for hypodontia provide good quality information to support decision making?

## Results

### **The use and perceived quality of information resources by dentists**

The questionnaire response rate was 49% (319/649). Twenty-two people declined participation of whom 13 provided reasons: retirement (4), no longer at address (6), maternity leave (1), non-clinical or non-UK dentist (2). Response rates varied by specialty: orthodontists (56%), hypodontia clinics (55%), paediatric dentists (46%), general dental practitioners (44%), restorative dentists including prosthodontists (32%). Most were NHS or mixed NHS/private dental practitioners, working in primary and/or secondary care with a range of job titles (Table 1). Ninety-one per cent of respondents examined and/or treated individuals with hypodontia. In line with expected job roles, general dentists examined and referred patients for treatment, hypodontia clinic dental professionals mainly advised and/or planned treatment, and specialists delivered treatments including orthodontic treatment to redistribute or eliminate spaces, tooth replacement with tooth or implant-supported prostheses and restorative camouflage. Most reported provision of verbal information, with variability in the provision of written or online information (Table 2). There was a clear difference in information provision by general dentist compared to specialists, but not between specialists working in primary and secondary care.

### Dentists’ perception of current patient information resources

Survey respondents perceived a need for better quality patient information in leaflet and online form (Table 3). Dentists’ suggestions for improvements to patient resources included changes to the focus, content, presentation and location of information (Table 4).

### Objective quality assessment for hypodontia patient information resources

**Figure 1:**
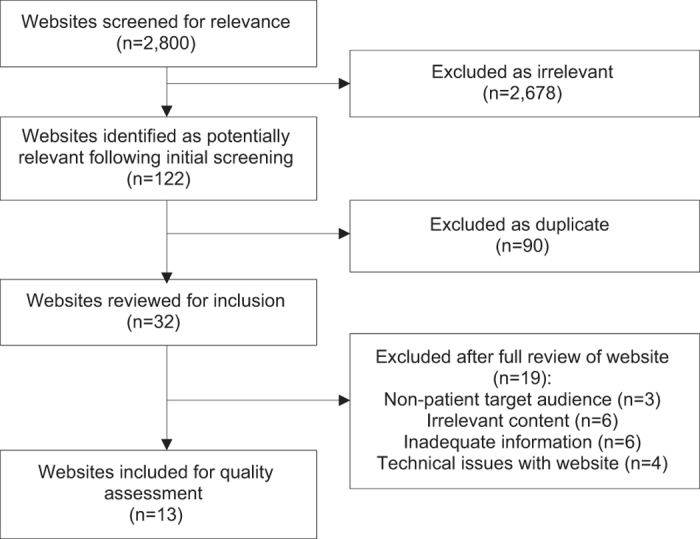
Selection process for online resources.

The survey respondents returned 18 PILs for inclusion in the quality assessment. From the systematic Internet search 122 potential relevant websites were identified; 90 were excluded as duplicates and nineteen were excluded after full review (Figure 1). Thirteen online resources fulfilled the selection criteria. The written and online resources were assigned a study number for inclusion in the quality assessment (Supplementary Table 2). Resources were coded by type of publisher, scope and purpose (Table 5).

Most PILs were published by dental charities or dental care providers; most online resources were developed by not-for-profit websites and blogs with unclear authorship and affiliation. The purpose of the information source was rarely stated explicitly and the lack of clarity led to challenges during quality assessment. The majority of the resources provided information to prepare people for consultation, a procedure, or aftercare following a specific procedure. One industry PIL for implant treatment (Resource 14) stated a role in decision-making but it was unclear what this meant. No resource aimed to provide information about patient experience, advocacy or engagement with dental professionals. The leaflets largely provided details about a specific treatment, while the online resources provided information about hypodontia as a condition.

The quality assessment for the PILs and online resources showed variation in scores for design, content and readability (Tables 6 and 7). For many resources, the lack of clarity about the purpose of the resource complicated assessment of the adequacy of the content, and the scores for coverage and comprehensiveness were frequently zero. No resources used methods to encourage patients to clarify what was important to them about their treatment and think about their own values. Few information sources employed techniques to aid literacy, such as flow diagrams and alternative explanations for numerical values and risk. No resources stated their sources of evidence and concerns about bias in information provision were noted in a number of resources, with particularly high bias in industry PILs (Resources 14 & 15). Web blogs and Not-for-Profit sites tended not to give details about authorship, credibility and evidence sources. The majority of readability scores indicated a reading ability of 7th Grade (12–13 years old) to 10th Grade (15–16 years old) was necessary to understand the resources; two online resources required a higher level of reading ability, equivalent to degree level education.

## Discussion

This research used mixed methods to identify and evaluate the quality of information for people with hypodontia that is provided by dental professionals and available on the Internet. The findings show there is considerable variability in the provision and quality of written information. This is consistent with studies in other areas of dentistry, where variability in comprehensiveness, transparency and readability were common problems.^4–10^ Challenges to information provision are likely to be compounded for interdisciplinary conditions such as hypodontia, where information needs to cover a range of complex interventions. No previous studies were identified that assessed provision of patient information in similar interdisciplinary areas of dentistry to enable comparison. One study assessing patient knowledge of dental implants found dentists are still the main source of information,^22^ a finding supported by a study measuring information-seeking in orthodontic patients.^23^ Interestingly the latter study, undertaken in 2013, found Internet use was low (8%).

Dental professionals recognised a need to provide better quality written information to support people with hypodontia. The quality assessment indicated most resources focused on information to prepare people for a consultation or specific procedure. No resources used techniques known to increase lay understanding or encourage evaluation of patient values and reasoning about treatment in the context of their lives. These findings imply current information provision is unlikely to improve dental literacy or support dental professionals in engaging their patients in making informed decisions about their care.

The study findings have informed specific recommendations for future improvements to resources to make information more patient-centred (Table 8). Information resources need to be clear in their purpose and target audience to ensure the content is sufficiently comprehensive to fulfil this role. The content of these patient resources described factors that dental service providers see as essential in patient care. Certainly, the suggestions for improving patient resources offered by the dental professionals focused on more comprehensive clinical information about treatment, with little emphasis on experience of care and quality of life. It is likely that the ‘professionalisation’ that occurs during training moves people from layperson to dental expert leading to changes in perception of the impact of oral symptoms, treatment needs and access to care.^3^ Patient information resources aim to address the inequality in professional and lay understanding of health conditions, but to do this, appreciation of the how people think about dental health and experience hypodontia and its management is essential. Adopting a co-production approach with people affected by hypodontia before and during the design of information reosurces may help address some of the limitations of current resources.

Future patient resources should consider incoporating tools that have been shown to proactively support better thinking by patients and enhance health literacy, treatment decision making and engagement with care (see DISCERN^19^ and IPDAS^20^). Guidance highlights how clinicians can make information balanced, non-directive and able to support people to consider their own values and preferences for treatment.^24^ Images and visual aids have a role in helping people understand numerical data, processes and technical procedures for those with low literacy.^25^ However, there is little evidence that dental images facilitate engagement, understanding, decision making, and adherence. It may be that in this clinical context, dental images may trigger an adverse emotional response and patient reaction.^26,27^

The increasing availability and use of multimedia and online information in health care is likely to continue changing how patients access information in the future. No respondents indicated use of a tablet device to share information but this is a potential future development, alongside information provision via mobile telephone apps. A randomised controlled trial is in progress in the UK to determine whether a patient information app for hypodontia will make patients better informed and more satisfied with the consultation process.^28^ Online information has scope for increasing the amount of information available while enabling tailoring for different severities and presentations of conditions and different treatment options. With this potential abundance of information it is essential that authorship and affiliations are transparent, information sources are cited to demonstrate credibility and information is kept up to date. In addition, there is an opportunity to develop components of information that are associated with better dental literacy and engagement with hypodontia management.

The survey successfully identified a number of resources that were unknown to the authors and although inclusion of resources for quality assessment was not exhaustive, it is unlikely inclusion of further resources would significantly affect the overall conclusions of the study. The response rate from the survey was relatively low (49%) although this is concordant with other postal surveys. There did not appear to be a systematic difference between responders and non-responders. It is unlikely that the results would have been significantly changed by a higher responses rate, as there was evidence of agreement in the opinions of the dentist groups. Only one questionnaire was sent to each respondent so for clinicians working in both primary care and secondary care settings there was little scope to differentiate between information provision in each care setting. This may have led an erroneous assumption that information provision was the same for clinicians across different settings.

The quality assessment tool was informed by established methods of assessing information quality and although not validated, it enabled systematic elicitation of information to enable a synthesis of current resources. The scope of this study was limited to information gathering and opinion from a dental professional perspective only. The findings from all three analyses suggest confidence in our findings that current resources are not able to boost patients’ thinking about hypodontia care and management.

## Conclusions

Information resources for people with hypodontia are limited in their scope, with most aiming to prepare people for consultation or a specific treatment but few providing an aid to decision making. Inadequacies in the design, content and readability of available resources prevent adequate knowledge transfer to enable patients to understand their condition and treatment. This implies current information provision is unlikely to improve dental literacy or support dental professionals in engaging people with hypodontia in making informed decisions about their care.

## Publisher’s note

Springer Nature remains neutral with regard to jurisdictional claims in published maps and institutional affiliations.

## Figures and Tables

**Table 1:** Demographics of participants in relation to service factors

*Dental Professional Group*	*Job title*^a^		*Care setting*		*Remuneration*	
Orthodontists (*n*=86^b^)	Specialist	64	Primary	50	NHS	26
	Consultant	24	Secondary	17	Private	1
	University	2	Both	19	Mixed	59
	DWSI	6				
Paediatric Dentists (*n*=100)	Specialist	43	Primary	23	NHS	90
	Consultant	39	Secondary	63	Private	4
	University	22	Both	11	Mixed	6
	SDO	2	Tertiary	2		
	Management	6	University	1		
Restorative Dentists (including prosthodontists) (*n*=29^b^)	Specialist	15	Primary	6	NHS	11
	Consultant	14	Secondary	12	Private	9
	University	3	Both	10	Mixed	9
	DWSI	3	University	1		
	Management	1				
GDPs (*n*=82)	Dentist	82	Primary	75	NHS	26
	Dentist with Special Interest	2	Secondary	6	Private	9
	Teaching role	7	Both	1	Mixed	47

a Each participant may have more than one job title.

b Includes dental professionals responding from the contact via the hypodontia clinic.

**Table 2:** Provision of information

*Dental professional group*	*Verbal*	*Written*	*Online*
Hypodontia clinics (*n*=11)	11 (100%)	10 (90%)	0
Orthodontists (*n*=81)	81 (100%)	51 (63%)	18 (22%)
Paediatric dentists (*n*=85^a^)	82 (96%)	21 (25%)	10 (12%)
Restorative dentists (*n*=23)	23 (100%)	17 (74%)	7 (30%)
General dental practitioners (*n*=69^a^)	67 (97%)	13 (19%)	5 (7%)
TOTAL (*n*=269)	264 (98%)	112 (42%)	40 (15%)

a Participants who do not manage people with hypodontia were excluded.

**Table 3:** Opinion regarding current information resources

*Question*	*Response*	*Hypo.(*n*=11)*	*Ortho.(*n*=81)*	*Paed.(*n*=85)*	*Rest.(*n*=23)*	*GDPs (*n*=69)*	*TOTAL (*n*=269)*
Would having further information for patients about hypodontia be useful?	Yes	10 (91%)	73 (90%)	82 (96%)	19 (83%)	63 (91%)	247 (92%)
	No	1 (9%)	8 (10%)	3 (4%)	4 (24%)	6 (9%)	22 (8%)
Do you think current sources of information for patients with hypodontia are fit for purpose?	Yes	3 (27%)	23 (28%)	7 (8%)	9 (35%)	8 (12%)	50 (19%)
	No	0	21 (26%)	20 (24%)	3 (18%)	7 (10%)	51 (19%)
	Unsure	8 (73%)	36 (44%)	57 (67%)	11 (47%)	53 (77%)	165 (61%)
	N/a	0	1 (0%)	1 (1%)	0	1 (1%)	3 (1%)
What would be your preferred method for providing patients with information about hypodontia (may be more than one per participant)?	Verbal	8 (73%)	50 (62%)	54 (64%)	17 (74%)	43 (62%)	172 (64%)
	Written	10 (91%)	67 (83%)	75 (88%)	23 (100%)	59 (86%)	234 (87%)
	Internet	8 (73%)	51 (63%)	60 (71%)	12 (71%)	39 (57%)	170 (63%)
	YouTube	1 (9%)	13 (16%)	12 (14%)	1 (6%)	16 (23%)	43 (16%)
Would you direct patients to a web-based information resource if the information was good quality?	Yes	11 (100%)	81 (100%)	83 (98%)	23 (100%)	67 (97%)	265 (99%)
	No	0	0	2 (2%)	0	2 (3%)	4 (1%)

**Table 4:** Dental professionals’ recommendations for improving patient information

*Theme*	*Recommendation*
Tailored information	Patient-centered Age-specific Information for different severities and presentations of hypodontia Restorative-led rather than orthodontic-led Local information linked to national information
Content of information	Information about treatment timing Information about treatment outcome – appearance, longevity, long-term costs Restorative treatment options (recommendation from orthodontists) Information about genetics and inheritance for family
Presentation of information	Use of images to illustrate options Before and after images Online resources with links between condition and examples of treatment options and outcomes Examples of other useful information resources given
Distribution of information	Online and interactive Online resources linked to dental charity websites (e.g. BOS, BSPD) Specific leaflet

**Table 5:** Purpose and scope of information sources

	*Number*		*Resource identifier*
	*PILS (*n*=18)*	*Online (*n*=13)*	
*Type of publisher*			
Dental charity	8	1	1,4, 5, 7, 10, 11, 17, 18, 29
			
*Care provider*			
Primary	5	2	3, 6, 8, 9, 16, 20, 27
Secondary	3	2	2, 12, 13, 19, 21
Industry	2	1	14, 15, 31
Not-for-profit	0	3	22, 24, 30
Blog	0	4	23, 25, 26, 28
			
*Purpose of information*			
Preparation for consultation/procedure	13	13	1-5, 10-13, 15-31
Aftercare to aid adjustment or coping	4	0	6- 9
Decision-making	1	0	14
Patient experience/advocacy	0	0	
			
*Scope of resource*			
Condition, service and treatment	2	11	1, 3, 20-30
Condition only	0	0	
Service only	1	1	2, 19
Treatment only	15	1	4-18, 31

**Table 6:**
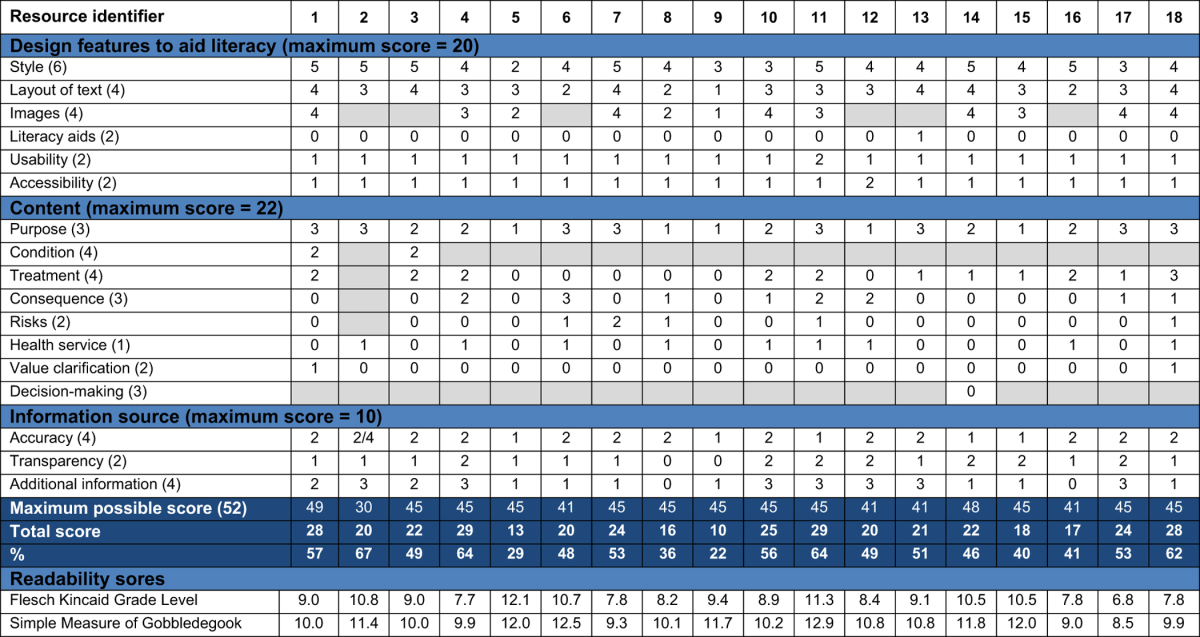
Quality assessment for the patient information leaflets (PILs)

**Table 7:**
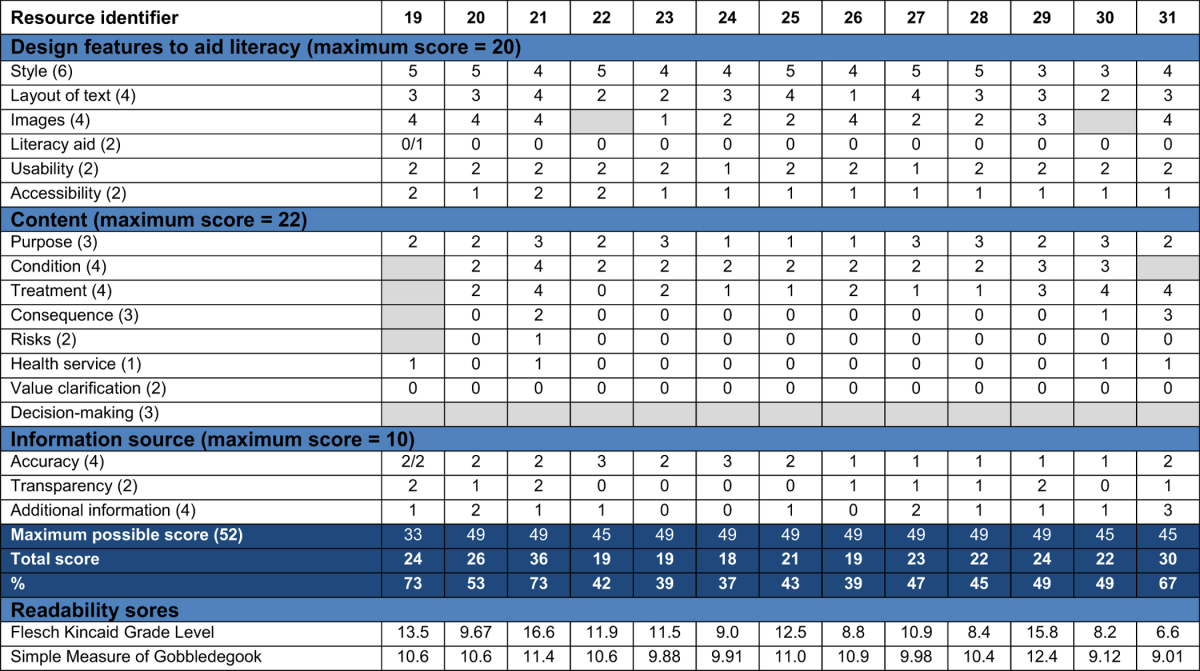
Quality assessment for the online resources

**Table 8:** Recommendations for improving patient information resources for hypodontia (* indicates recommendations arising from best practice guidance)

*Role of information resource*
The purpose of information and target audience should be explicitly stated
People affected by hypodontia should be involved in directing the co-development of resources that fulfil information needs*
Resources aiming to support decision-making should include:
An explicit acknowledgement of the decision to be made
Factors to be considered
Methods to clarify values during decision-making
Value clarification methods should be used to encourage patients and families to interpret information with consideration of their own values

*Comprehensiveness of content*
Information about the impact and timeline of hypodontia should be included
Information about hypodontia should be included in resources aimed at management to ensure adequate background understanding
Information about treatment should emphasise who treatment is suitable for, alternative treatment options including no treatment and provide an explanation of how treatment works.
The description of the consequence of treatment should include:
Quantification of effectiveness (success/ failure of treatment)
All side effects relevant to the treatment/ procedure described
Impact of treatment on life, both positive and negative
Risks should be described for all side effects and potential harms using more that one numerical or visual method*
Information about the health service should be included, particularly where more than one option for service delivery exists

*Design*
Features should be selected to optimise design:
Use of non-white pale background & matt paper*
Avoidance of underlining, italics and block capitals*
Use of larger font in bold or boxes/borders for emphasis*
Use of navigation aids to improve usability*
Images should have a clear purpose and include an explanatory caption
Users should be involved in selecting images that are most useful*
Consideration should be given to including literacy aids, for example:
Descriptions for numerical data*
Flow diagrams for processes*
Diagrams for technical procedures*
Users should be involved in developing literacy aids*

*Accessibility*
Tailored resources should be available to increase accessibility*
Readability needs to be improved to ensure resources are accessible to lay people with moderate-low reading ability

*Credibility*
Authorship and credentials should be explicitly stated, particularly for online resources
Sources of evidence should be provided, particularly areas where a lack of evidence contributes to uncertainty
