# Supplementary material for: Assessment of information resources for people with hypodontia
Source: BDJ Open. 2018 Mar 9;4:18001–. doi: 10.1038/bdjopen.2018.1 (PMC5844244; doi:10.1038/bdjopen.2018.1)
Supplement: Supplementary Information [file bdjopen20181-s1.docx]

**Supplemental tables**

Table 1: Results from the systematic search using online search engines

| **Search term** | **Search engine** | **Total number of hits** | **Included for review following screening of first 100 hits** |
| --- | --- | --- | --- |
| Hypodontia | Google | 131,000 | 13 |
|  | Bing | 101,000 | 16 |
|  | Yahoo | 98,500 | 15 |
|  | YouTube | 187 | 0 |
| Developmentally missing teeth | Google | 220,000 | 5 |
|  | Bing | 23,300,000 | 6 |
|  | Yahoo | 23,300,000 | 6 |
|  | YouTube | 253 | 0 |
| Congenitally missing teeth | Google | 102,000 | 9 |
|  | Bing | 508,000 | 9 |
|  | Yahoo | 508,000 | 9 |
|  | YouTube | 1,390 | 0 |
| Absent teeth | Google | 25,900,000 | 11 |
|  | Bing | 22,100,000 | 8 |
|  | Yahoo | 22,100,000 | 8 |
|  | YouTube | 3,510 | 0 |
| Tooth dental aplasia agenesis | Google | 267,000 | 1 |
|  | Bing | 54,100 | 3 |
|  | Yahoo | 53,400 | 3 |
|  | YouTube | 1,210 | 0 |
| Gappy teeth | Google | 61,100 | 0 |
|  | Bing | 28,700 | 0 |
|  | Yahoo | 30,400 | 0 |
|  | YouTube | 2,020 | 0 |
| Spaced teeth | Google | 1,140,000 | 0 |
|  | Bing | 28,300,000 | 0 |
|  | Yahoo | 27,600,000 | 0 |
|  | YouTube | 5,470 | 0 |
|  |  |  | **122** |

Table 2: List of patient information resources that were included for quality assessment (*anonymised)

| **Patient Information Leaflets (PILs) (n=18)** | |
| --- | --- |
| American Association of Orthodontists | “All about Orthodontics” |
| Association of Dental Implantology | “Considering Dental Implants?” |
| Bicon | “Enjoy the confidence of beautiful teeth” |
| British Dental Health Foundation | “Tell me about Implants”  “Tell me about Orthodontic Treatment”  “Tell me about Bridges and Partial Dentures” |
| British Orthodontic Society | “Fixed appliances”  “Hypodontia”  “Tooth transplantation” |
| Dental Practices* | “Dental Implants”  “Hypodontia (Missing Adult Teeth)” |
| NHS Hospital Trusts* | “Dental Implants – Dental Services Information for Patients”  “Hypodontia Clinic – Information for patients”  “Insertion of intraoral implant” |
| Nobel Biocare | “Complete Yourself” |
| Specialist Orthodontic Practices* | “Fixed Orthodontic Appliances”  “Orthodontic Treatment”  “Your fixed braces” |
| **Online resources (n=13)** | |
| \| [www.bartshealth.nhs.uk/our-services/services-a-z/d/dental/for-patients/hypodontia/](http://www.bartshealth.nhs.uk/our-services/services-a-z/d/dental/for-patients/hypodontia/)  [www.braces4oxford.co.uk/MissingTeeth.htm](http://www.braces4oxford.co.uk/MissingTeeth.htm)  [www.bupa.co.uk/health-information/directory/t/teeth-replacing](http://www.bupa.co.uk/health-information/directory/t/teeth-replacing)  [www.deardoctor.com/inside-the-magazine/issue-17/when-permanent-teeth-do-not-grow/](http://www.deardoctor.com/inside-the-magazine/issue-17/when-permanent-teeth-do-not-grow/)  [www.drtreys.com/congenitally-missing-teeth.php](http://www.drtreys.com/congenitally-missing-teeth.php)  [www.gotoapro.org/missing-toothteeth/](http://www.gotoapro.org/missing-toothteeth/)  [www.healthtipsandguides.net/hypodontia-absent-teeth.htm](http://www.healthtipsandguides.net/hypodontia-absent-teeth.htm)  [www.hypodontia.com](http://www.hypodontia.com)  [www.missingteeth.co.uk](http://www.missingteeth.co.uk)  [www.oralanswers.com/congenitally-missing-teeth-treatment/](http://www.oralanswers.com/congenitally-missing-teeth-treatment/)  [www.todaysorthodontist.com/hypodontia/](http://www.todaysorthodontist.com/hypodontia/)  [www.uclh.nhs.uk/OurServices/ServiceA-Z/EDH/RESDEN/EDHHC/Pages/Home.aspx](http://www.uclh.nhs.uk/OurServices/ServiceA-Z/EDH/RESDEN/EDHHC/Pages/Home.aspx)  en.wikipedia.org/wiki/Hypodontia \| \| --- \| | |
